# Moral Disengagement, Social Norms, and Motivational Profiles Influence Attitudes Toward Doping Among Spanish Athletics Coaches

**DOI:** 10.3389/fspor.2022.842959

**Published:** 2022-03-11

**Authors:** Elena García-Grimau, Ricardo De la Vega, Arturo Casado

**Affiliations:** ^1^Department of Physical Education, Sport and Human Movement, Universidad Autónoma de Madrid, Madrid, Spain; ^2^Spanish Agency for Health Protection in Sport, Madrid, Spain; ^3^Centre for Sport Studies, Rey Juan Carlos University, Madrid, Spain; ^4^Faculty of Health Sciences, Isabel I University, Burgos, Spain

**Keywords:** anti-doping, doping, moral disengagement, social norms, coaches, competitive sport, athlete support personnel, motivational profile

## Abstract

Coaches strongly influence athletes' attitudes toward doping and can shape athlete's beliefs, behaviors, and decisions to be for or against doping. Coached-centered studies examining multiple factors affecting coaches' doping attitudes and behavior are scarce. The aim of this study was to analyze for the first-time attitudes toward doping in athletics coaches using the Sport Drug Control Model (SDCM) as a theoretical framework. A secondary aim was to determine the factors in the model predicting attitude and susceptibility toward doping. A cross-sectional study was carried out using a sample consisting of 201 Spanish athletics competitive level coaches from whom 11.4% were female. Participants completed a cross-sectional online survey. Structural equation modeling showed a good fitness of the SDCM. Positive attitudes toward doping predicted high susceptibility to doping (β = 0.39, *p* < 0.001). Moral disengagement (β = 0.58, *p* < 0.001), descriptive norms (β = 0.42, *p* = 0.001), ego-oriented goals (β = 0.34, *p* < 0.05), and self-efficacy to refrain from doping (β = 0.26, *p* < 0.05) displayed a significant influence on attitudes toward doping. Self-reported doping prevalence in coaches was 4.5%. These variables should be considered when designing anti-doping research projects and educational programs aiming at modifying coaches' attitudes toward doping. It is recommended to focus more efforts on coaches, without putting aside the athletes, and therefore turn coaches into reliable doping prevention factors. To this end, it is necessary to enhance scientific research and then develop, implement, and promote more educational programs targeting coaches, on a mandatory basis while covering the specific needs of coaches so that they can perform their role as anti-doping educators in an effective, committed, and proactive manner.

## Introduction

Coaches strongly influence athletes' attitudes toward doping and can shape athlete's beliefs, behaviors, and decisions to be for or against doping (Barkoukis et al., 2019; García-Grimau et al., 2021). The impact of the athlete's entourage on attitudes toward doping has been reported in different studies (Backhouse and McKenna, 2012; Mazanov et al., 2014; Engelberg and Moston, 2016) and the coach is considered part of the Athlete Support Personnel (ASP) as well as parents/guardians, physiotherapists and other professionals supporting and working directly with athletes, as defined by the World Anti-Doping Code (WADC) (World Anti-Doping Agency, 2021a). Some specific roles and responsibilities of the ASP are the following: cooperating with anti-doping organizations, complying with anti-doping regulations that are applicable to them or their athletes, and using their influence on athletes' values and behavior to foster anti-doping rules (World Anti-Doping Agency, 2021a). Coaches could engage on doping behavior, act as social facilitators in doping in sport (Vakhitova and Bell, 2018) and incite their athletes to commit anti-doping rule violations (ADRVs) (i.e., those displayed in articles ranging from 2.5 to 2.11 of the Code). In these cases, coaches would have conflicted with their responsibility of encouraging their athletes to avoid the use of doping. According to the current anti-doping legislation (World Anti-Doping Agency, 2021a), seven ADRVs are applicable to ASP: tampering or attempted tampering, possession of a prohibited substance or method, trafficking or attempted trafficking, administration, or attempted administration to any athlete of any prohibited substance or method, complicity, or attempted complicity, prohibited association, and acts to discourage or retaliate against reporting to authorities. These non-analytical ADRVs are typically reported in this population. For example, 16 sanctions were imposed worldwide on ASP in 2018 (World Anti-Doping Agency, 2020) and 155 members of ASP are currently serving a period of ineligibility (World Anti-Doping Agency, 2021b).

Beyond the legislative framework, different studies reported a lack of anti-doping knowledge in coaches (Mazanov et al., 2014; Morente-Sánchez and Zabala, 2015; Engelberg and Moston, 2016). Other studies analyzed their beliefs about threats to health (Scarpino et al., 1990; Laure et al., 2001) and their attitudes toward doping and doping prevention. Fung and Yuan (2006) examined perceived knowledge, actual knowledge, attitudes, subjective norms, and behavior in relation to doping and anti-doping in sport in 114 coaches through the conceptual framework of the theory of Planned Behavior (Ajzen, 1991). Results revealed a negative correlation between the perceived and actual knowledge, which implies that coaches may under-estimate or over-estimate their knowledge regarding performance enhancing drugs/substances (PES) and doping control. This finding reflects the complexity of the phenomenon of doping in sport (Fung and Yuan, 2006). The authors suggest the need to design mandatory educational programs specifically designed for coaches. Furthermore, coaches are typically aware that they have an important role in doping prevention and generally display negative attitudes toward doping (Backhouse et al., 2015). Nevertheless, they are not fully committed to the prevention of doping (Patterson et al., 2014; Engelberg et al., 2019), and they need more resources and support to be able to proactively prevent doping among their athletes (Barkoukis et al., 2019).

Attitudes toward doping represents a strong predictor of doping susceptibility and behavior (Gucciardi et al., 2010; Barkoukis et al., 2013; Blank et al., 2016; García-Grimau et al., 2020). However, studies analyzing factors affecting coaches' doping attitudes and behavior are scarce. Nonetheless, a few psychological factors among coaches which may influence the use of doping in their athletes have been highlighted. Sullivan et al. (2015) explored the doping confrontation efficacy in coaches through the Doping Confrontation Efficacy Scale. They reported that coaches who are more prone toward task-involved climates tend to show higher efficacy on confronting athletes, thus preventing them from doping use (Sullivan et al., 2015). Findings from a cluster randomized controlled trial reported that coaches adopting a motivational climate reduced athlete willingness to dope (Ntoumanis et al., 2021). In addition, moral disengagement was strongly correlated with doping attitudes and intentions toward doping in athletes (Kavussanu et al., 2019; García-Grimau et al., 2021) and plays an important role in coaching style and climate (Hodge and Lonsdale, 2011; Chen et al., 2017). Moreover, recent qualitative evidence (Patterson and Backhouse, 2018; Barkoukis et al., 2019) reported that displacement and diffusion of responsibility, which are considered two of the mechanisms involved in the moral disengagement construct (Bandura et al., 1996), influence coaches' opinions and behavior toward doping. Coaches delegate their role in anti-doping prevention on authorities, which reflects an unwillingness to proactively engage in anti-doping education; furthermore, Barkoukis et al. (2019) reported that the stigmatization of doping behavior prevents athletes and coaches from being effectively educated about doping. Horcajo and De La Vega (2016) carried out an experimental study in which the conviction about attitudes related to doping and the effect of deliberative thinking (i.e., high- vs. low-elaboration likelihood) on that conviction were analyzed in soccer coaches. They reported that coaches with high elaboration likelihood showed more conviction about their attitudes than those with low elaboration likelihood. Morente-Sánchez and Zabala (2015) examined knowledge, attitudes, and beliefs toward doping in 237 soccer technical staff (including 101 coaches) and highlighted their lack of doping-related knowledge.

Overall, coaching style and climate, and psychological and moral factors have been quantitatively and independently analyzed in the doping literature to finally understand their relationships with doping attitudes in athletes. However, studies examining a large number of factors influencing coaches' doping attitudes and behavior under a specific theoretical framework are scarce. Literature reviews carried out by Backhouse et al. (2007, 2015) confirmed the lack of quantitative research guided by a theoretical framework on doping attitudes and behavior in coaches. The Sport Drug Control Model (SDCM) was developed by Donovan et al. (2002) and incorporates different frameworks from the behavioral sciences such as the Theory of Reasoned Action (Fishbein, 1979) and the Theory of Planned Behavior (Ajzen, 1991). The SDCM analyzes several factors influencing athletes' attitudes toward and susceptibility to doping: morality, legitimacy, benefits and threat appraisals, motivational profiles, beliefs about reference groups' endorsement of doping methods/substances, use of legal supplements, beliefs about the availability of PES and relevant authorities' control over trafficking of doping methods/substances, beliefs about the affordability of doping methods/substances, attitudes toward doping, susceptibility to doping, and self-reported use of banned PES or methods (PESM). The World Anti-Doping Agency integrated this model as a guideline for anti-doping organizations (World Anti-Doping Agency, 2015) and it has been tested in athletes (Gucciardi et al., 2010; Jalleh et al., 2013; García-Grimau et al., 2021) showing validity and reliability. Jalleh et al. (2013) and García-Grimau et al. reported that the most influential factors were morality, reference group opinion, and legitimacy. Moreover, Donovan et al. (2002) observed that the SDCM could be adapted for application to ASP but has not yet been applied to coaches. In this study, the SDCM is applied for the first time in coaches with the aim of assessing the reproducibility of the model in ASP. Secondary study purpose was to determine the factors in the SDCM that most influence coaches' attitudes and susceptibility toward doping, and their doping prevalence.

## Materials and Methods

### Participants and Design

Athletics is the third summer Olympic sport most affected by doping, reporting 15% of the total ADRVs worldwide in 2018 (World Anti-Doping Agency, 2020). However, to the best of the authors' knowledge no study analyzing attitudes and behavior toward doping in Spanish athletics coaches has been conducted previously. Thus, athletics coaches were recruited between February and March 2021 to participate in a cross-sectional online survey *via* e-mail from the database of the National School of Coaches of the Royal Spanish Athletics Federation. The questionnaire was sent to 1,432 coaches of whom 163 completed the survey. To achieve a suitable statistical sample size for structural equation modeling (SEM) (Tabachnick and Fidell, 2013), 38 athletics coaches were further online recruited *via* e-mail from the Athletics Federations of the Spanish regions of Madrid, Aragón, and Navarra in April 2021. In the first section of the online survey, coaches received information explaining the aims and procedures of the study and consent to take part. Participants were reassured about the anonymity and confidentiality of their responses and about their right to withdraw at any time. A final sample of 201 Spanish athletics coaches successfully completed the survey, from whom 62.2% were aged between 30 and 59 years and 88.6 and 11.4% were male and female, respectively. Coaches were specialized in middle- and long-distance running (38.8%,), sprinting/hurdle (26.9%), jumping/throwing (19.9%), combined events (10.0%), and race walking (4.5%). Regarding the level of performance achieved by their athletes, coaches were training an athlete who had participated at least once in Olympic Games (9.5%), World Athletics Championships (11.9%), European Athletics Championships (10.0%), other international events with the national team (15.4%), national Athletics Championships (45.8%), and regional Championships (7.5%).

### Instrument

The Spanish SDCM questionnaire which was previously used on athletes and provided validity and reliability (García-Grimau et al., 2021), has been adapted for coaches to measure the following constructs: (1) moral disengagement; (2) benefits appraisal; (3) threat appraisal; (4) self-efficacy to refrain from doping; (5) goal orientations; (6) subjective norms: reference groups' endorsement of doping methods/substances; (7) descriptive norms: belief of doping use in others; (8) attitudes toward doping, and (9) susceptibility to doping. The questionnaire measures are described as follows.

#### Moral Disengagement

Morality construct was measured with the Moral Disengagement in Doping Scale, which has shown good internal consistency, reliability, and validity (Kavussanu et al., 2016). Coaches were asked to indicate their level of agreement with six statements measured on a Likert scale ranging from 1 (strongly disagree) to 7 (strongly agree). Example items are: “Athletes cannot be blamed for doping use if their teammates pressure them to do it” and “Athletes should not be blamed for doping use if everyone is doing it.”

#### Benefits Appraisal

In line with previous research (Jalleh et al., 2013; García-Grimau et al., 2021), benefit appraisal is measured in terms of (1) perceived performance-enhancing effects of banned substances and methods use and (2) likelihood of potential positive outcomes for performing well in sport. Questions were reformulated to adapt them to coaches. For example, to assess (1), participants were asked to rate from definitely would not (1) to definitely would (5) “If any of your athletes were to use a banned PESM of his/her choice, how likely is it that he/she would improve his/her performance?” To assess (2) participants were asked “To what extent does your sport offer you these outcomes if your athletes perform well?” and rate from a lot (1) to not at all (3) six answer-items (i.e., national celebrity status, future financial security).

#### Threat Appraisal

Threats relating to (1) deterrence and (2) ill-health effects were measured. To assess (1) coaches were asked two questions to measure their perceived likelihood of an athlete being tested in and out of competition, and of evading detection if using doping in and out of competition, using a 5-point scale ranging from (1) very likely to (5) not at all likely. To assess (2) participants were asked to score the harm level of six different PESMs using a 5-point scale from 1 (a lot of harm) to 5 (no harm).

#### Self-Efficacy to Refrain From Doping

To assess coaches' ability to avoid the use of PESMs within their athletes or resist doping temptation, the ten-item Doping Self-efficacy scale (Lucidi et al., 2008) was used and adapted to coaches (i.e., “to avoid using PESMs with my athletes before a competition even when I know I can get away with it,” “to resist the temptation to use PESMs with my athletes to improve their performance”). Participants were asked to rate from completely capable (1) to not at all capable (7).

#### Goal Orientation

Research indicates that ego-oriented goals increase doping use likelihood (Ring and Kavussanu, 2018a) while task-oriented goals are related to lower susceptibility to doping (Ntoumanis et al., 2021). Coaches were asked to indicate their level of agreement with six statements from the ego-oriented subscale (i.e., “I am the best,” “I show other people I am the best”) using a five-point Likert scale from strongly disagree (1) to strongly agree (5).

#### Subjective Norms

In line with previous research (García-Grimau et al., 2021) coaches' perceptions of others' attitudes toward doping were assessed with the following question: “If any of your athletes decided to use a PESM, to what extent do you think each of the following people would approve or disapprove or would not care either way if they did that?” Six-response items were presented to participants (i.e., parents, teammates, sport doctors, and manager) and asked them to rate from would definitely approve it (1) to would definitely disapprove it (5).

#### Descriptive Norms

To assess coaches' beliefs regarding others' use of doping, they were asked to indicate the percentage of perceived doping prevalence in five statements (i.e., “Out of 100%, how many athletes in your sport do you believe engage doping to enhance their performance,” “Out of 100%, how many coaches in your sport do you believe would encourage their athletes to use doping to enhance their performance?”).

#### Attitudes Toward Doping

Following the work of Petróczi (2002) a single-item was used to measured coaches' attitudes toward the use of PESM: “In your sport, how necessary do you believe it is for athletes to use banned performance-enhancing substances at least at some time, to perform at the very highest levels?” Responses were rated on a Likert scale ranged from 1 (definitely have to use) to 5 (definitely don't have to use).

#### Susceptibility to Doping

Susceptibility to doping is measured using a hypothetical scenario adapted from previous research (Bamberger and Yaeger, 1997; García-Grimau et al., 2021). Coaches were asked to imagine a situation to use a PESM with their athletes to enhance their performance. The scenario is described below:

“If you were offered a banned PES under medical supervision at low or no financial cost and the banned PES could make a significant difference to your athletes' performance and was currently not detectable, how much consideration do you think you might give to this offer?”

Responses were rated from not at all consideration (1) to a lot of consideration (4).

#### Doping Prevalence

Doping prevalence among coaches is measured in terms of self-reported administration or attempted administration to athletes of a PESM (lifetime or in the last 12 months). For the lifetime doping prevalence, participants were presented with seven items/statements and told to indicate which one of the statements most applies to them. Each item was scored from 1 (I have never considered using a banned PESM with my athletes) to 7 (I regularly try or use banned PESM with my athletes). This variable was transformed in a dichotomous variable range from 0 (never use PESM) to 1 (ever use). For the prevalence of doping in the last 12 months, coaches were presented with six different PESM and asked: “In the last 12 months, how often have you used any of the following PESM with your athletes, for whatever reason?” Responses were rated from 1 (have never used) to 6 (more than 10 times). This variable was transformed in a dichotomous variable range from 0 (never use PESM) to 1 (use 12 months). These two variables were combined and recoded into a single variable measuring total doping prevalence among coaches. This variable only measures one of the seven possible ADRV that ASP can commit (see introduction and WADC).

#### Indirect Doping Prevalence Among Athletes

Indirect doping prevalence among athletes were measured by asking coaches the following dichotomous question: “Have any of your athletes ever tested positive for a banned PES?”

### Protocol

Ethics committees from Isabel I de Castilla International University (UI1-PI016) and World Anti-Doping Agency (2019-A2) provided ethical approval for the completion of the present study. All the participants signed a consent form to participate in this study which was conducted in accordance with the Declaration of Helsinki. Athletes were informed about the aims and purposes of the study and reassured about their anonymity and confidentiality of their data.

### Data Analysis

Descriptive statistics, reliability, and internal consistency analysis of the study variables were performed through the Statistical Package for the Social Sciences (SPSS) version 24.0 (IBM, Armonk, NY, USA). Missing values were checked before statistical analysis. Missing data for each variable was low (i.e., 0.0–4.4%) and replaced through the expectation maximization method (Graham, 2009). Means (95% confident intervals [CI]), standard deviations (SDs), McDonald's ω, average variance extracted (AVE) and composite reliability (CR) were calculated as a measure of reliability and internal consistency. SEM was carried out to test the SDCM in coaches through AMOS package for SPSS version 24.0. An examination of the measurement portion of the model and setting constraints was made to avoid identification issues. To evaluate the adequacy of the model the fit indices recommended in guidelines (Hair et al., 2010; Tabachnick and Fidell, 2013) were employed: ratio of the χ^2^ to the degrees of freedom (χ^2^/df <2), comparative fit index (CFI > 0.9), Tucker Lewis Index (TLI > 0.9), root-mean-square error of approximation (RMSEA ≤ 0.08) and Standardized Root-Mean-Square Residual (SRMR ≤ 0.10).

## Results

**Figure 1:**
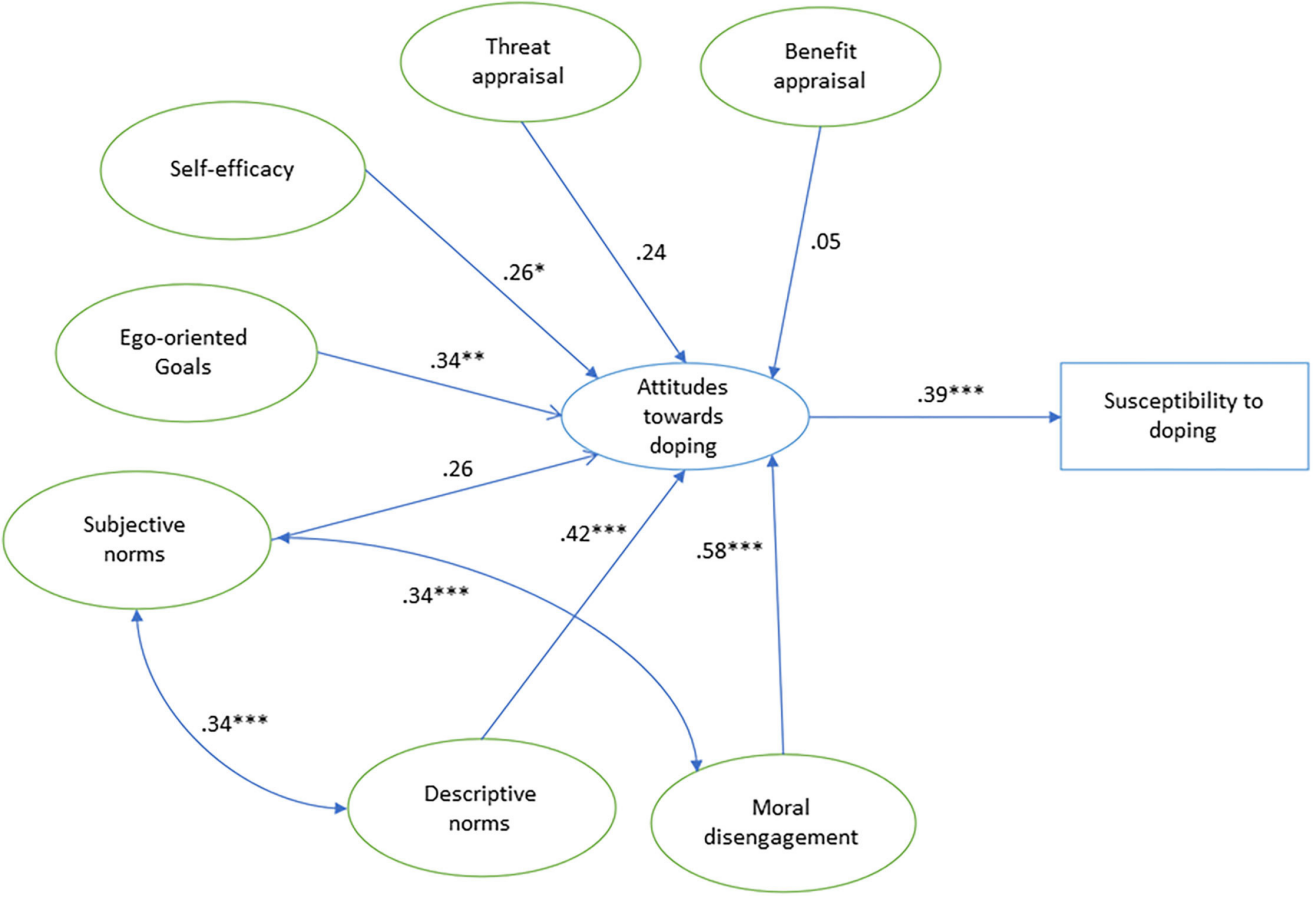
Overview of results of structural equation model analysis with standardized parameter estimates. Different levels of significance according to *p*-value: *p ≤ 0.05, **p ≤ 0.01, and ***p ≤ 0.001.

Descriptive statistics for the different variables analyzed (see Table 1) indicate that coaches reported on average negative attitudes toward doping and low levels of susceptibility to doping and moral disengagement. With respect to psychological factors, coaches stated on average a high self-efficacy to refrain from doping and moderate ego-oriented goals. Regarding social norms, they reported a high subjective norm. They believed that, on average, reference groups would disapprove doping behaviors. With respect to descriptive norms, coaches perceived an average doping prevalence of 19.5% (1.95 ± 1.74 [mean ± SD]) (see Table 2 for further details). Measures showed good internal consistency and reliability, with omega (ω) values > 0.6, AVE values > 0.4 and CR values > 0.7 (see Table 1). Self-reported doping prevalence among coaches was 4.5%, and 3% acknowledged having had an athlete who has tested positive for a prohibited substance. The SEM analysis of the SDCM in coaches (Figure 1) revealed a good fit of the data: χ^2^/df = 1.76, *p* < 0.001, CFI = 0.93, TLI = 0.96, RMSEA = 0.062 (90% CI = 0.054, 0.070), and SRMR = 0.09. Covariance between moral disengagement and subjective norms, and between subjective norms and descriptive norms did not change the model fitness and improved the standardized parameter estimates and significance. Among the standardized parameter estimates (Figure 1), all the relationships were significant excepting threat appraisal, benefit appraisal, and subjective norms. Moral disengagement (β = 0.58, *p* < 0.001) and descriptive norms (β = 0.42, *p* < 0.001) were the strongest predictors of attitudes toward doping in coaches. Regarding motivational and psychological profiles, ego-oriented goals (β = 0.34, *p* ≤ 0.01) and self-efficacy to refrain from doping (β = 0.26, *p* < 0.05) were also good predictors of coaches' doping attitudes, while ego-oriented goals displayed a greater level of significance and higher standardized parameter estimate than self-efficacy. Moreover, attitudes toward doping predicted doping susceptibility significantly (β = 0.39, *p* < 0.001).

**Table 1:** Descriptive statistics, reliability, and internal consistency estimates for the variables measuring the sport drug control model through structural equation modeling.

**Variables**	**Range**	**Mean (CI)**	**SD**	**ω**	**AVE**	**CR**
Susceptibility to doping	(1) not at all to (4) a lot of consideration	1.08 (1.04, 1.13)	0.32	–	–	–
Attitudes toward doping	(1) definitely don't have to use to (5) definitely have to use	1.67 (1.52, 1.81)	1.05	–	–	–
Moral disengagement	(1) Strongly disagree to (7) strongly agree	1.37 (1.28, 1.46)	0.66	0.68	0.48	0.77
Benefit appraisal	Performance enhancing effect: (1) would not to (5) definitely would	3.21 (3.06, 3.36)	1.07	0.89	0.59	0.89
	Positive outcomes: (1) a lot to (3) not at all	1.50 (1.45, 1.55)	0.34	0.72	0.48	0.82
Threat appraisal	Testing likelihood: (1) very likely to (5) Not at all likely	3.57	1.27	–	–	–
	Evading detection: (1) Very likely to (5) Not at all likely	2.83	1.20	–	–	–
	Ill-health effect: (1) A lot of harm to (5) no harm	2.07 (1.9, 2.24)	1.10	0.94	0.73	0.94
Motivational profiles: self-efficacy to refrain from doping	(1) completely capable to (7) Not at all capable	1.59 (1.37, 1.80)	1.54	0.98	0.84	0.98
Motivational profiles: ego-oriented goals	(1) strongly disagree to (5) strongly agree.	2.14 (2.03, 2.26)	0.83	0.82	0.53	0.87
Subjective norms: Reference Groups' Endorsement of Doping Methods/Substances	(1) would definitely approve to (5) would definitely disapprove	4.14 (4.04, 4.24)	0.74	0.88	0.61	0.95
Descriptive norms: perception of others' use of doping		19.5* (17.1, 22.0)	17.4	0.93	0.76	0.94

CI, confident intervals; SD, standard deviation; ω, McDonald's ω; AVE, average variance extracted; CR, composite reliability. *Average percentage of perceived doping.

**Table 2:** Descriptive norms: coaches' beliefs regarding others' use of doping.

**Statements**	**Mean (SE)**
Out of 100%, how many athletes in your sport do you believe engage doping to enhance their performance	20.0 (1.3)
Out of 100%, how many elite athletes in your country do you believe engage in doping to enhance their performance?	23.4 (1.6)
Out of 100%, how many elite athletes do you believe will be engaged in doping during the next 2 years to enhance their performance?	25.3 (1.7)
Out of 100%, how many coaches in your sport do you believe would encourage their athletes to use doping to enhance their performance?	12.8 (1.0)
Out of 100%, how many coaches in elite sports in your country do you believe would encourage their athletes to use doping to enhance their performance?	16.5 (1.4)

## Discussion

The main study objectives were to analyze attitudes and susceptibility toward doping in coaches while testing the SDCM for the first time in ASP, and to determine which factors were the strongest predictors of their doping attitudes. So far, the SDCM had been applied to athletes, its application in a new population is an added value to this research. The most important findings in regard to the relationship between the variables were that attitudes toward doping predicted high susceptibility to doping, and were highly influenced by moral disengagement and descriptive norms in athletics coaches. The observed strength of the relationship between attitudes toward doping and doping susceptibility agrees with results from other studies carried out in athletes (Barkoukis et al., 2013; Blank et al., 2016; García-Grimau et al., 2021). However, to the best of the authors' knowledge by the time of writing, this relationship was not previously tested in coaches and the SDCM was not ever adapted and examined in coaches, hence this study brings innovation to anti-doping research.

In addition, the fact that morality and more particularly moral disengagement was found to be the factor with the strongest influence on attitudes toward doping in coaches reveals that the more they are morally disconnected, the more their favorable attitudes toward doping. These results are in line with other studies in athletes (Jalleh et al., 2013; García-Grimau et al., 2021) and coaches (Patterson and Backhouse, 2018; Barkoukis et al., 2019) in which moral disengagement displayed a strong influence on doping likelihood acting as a direct predictor or mediator (Ring and Kavussanu, 2018b). The relevance of moral variables on doping prevention has been proven in empirical research. For example, a recent anti-doping interventional study which focused on developing morality in British and Greek athletes reported a reduction in the likelihood of doping use (Kavussanu et al., 2021). In this sense, morality variables should be considered when implementing educational programs targeting ASP and coaches in particular.

Social norms were measured in the present study through subjective and descriptive norms. Our results show that descriptive norms were a significant and positive predictor of attitudes toward the use of doping in coaches, which reflects that the perception of high prevalence of doping in others could enhance their attitudes toward doping, as they tend to normalize this behavior. Their perception of incidence of performance enhancing drug use across all sports and for athletics were 23.4 and 20.0%, respectively (see Table 2). These results are consistent with those by Moston et al. (2015b), who reported that doping prevalence perception in 92 Australian coaches across all sports was 20.9%. Additionally, coaches believe that 25.3% of athletes will be engaged in doping in the next 2 years.

Subjective norms (i.e., social approval of significant others) did not display a significant influence on doping attitudes, which contrasts with findings from other studies performed in athletes (Lazuras et al., 2010; García-Grimau et al., 2021). For example, a recent SDCM analysis in Spanish national standard and elite track and field athletes reported that moral disengagement and subjective norms were the variables with the greatest influence on attitudes toward doping (García-Grimau et al., 2021). Therefore, the absence of influence of this construct on coaches' doping attitudes, which contrasts with that in athletes, reflects their need to receive anti-doping education from sporting bodies and anti-doping organizations given their important role as anti-doping educators. Overall, this finding highlights that normative beliefs can be considered determinants of attitudes and behavior toward doping in the sport society.

Regarding motivational profiles, self-efficacy, and ego-oriented goals were significant predictors of coaches' doping attitudes in the present study. Coaches with ego-oriented goals and less ability to resist temptations may be more prone to doping. This finding is consistent with results from Matosic et al. (2016, 2020), who reported that coach narcissism is positively associated with controlling coaching behavior, and athletes' perceptions of coach behavior predict athletes' attitudes toward doping. Moreover, self-regulatory efficacy in coaches helps to create a motivational climate (Sullivan et al., 2015), reduce athlete willingness to dope (Ntoumanis et al., 2021) and, according to our findings, prevent them from displaying positive attitudes toward doping. Hence, those motivational factors are strong predictors of attitudes and susceptibility to doping in both coaches and athletes.

Benefit and threat appraisal factors did not significantly predict coaches' doping attitudes. That means that the potential benefits or positive outcomes that a coach could achieve by cheating, and the level of threat perceived by the coach due to deterrent effect or risk to health, are not significant factors influencing their attitudes toward doping. Moston et al. (2015a) analyzed the perceived incidence of deterrents on drug use in Australian athletes and coaches and reported that coaches saw deterrents as less credible than athletes, with a low perceived likelihood of detection. Similarly, 75% of the coaches who participated in our study perceived the likelihood of being tested out of competition as low (not at all likely or not likely) and generally considered that doping has positive effects on athletes' performance. However, the latter would not result in substantial economic benefits or positive outcomes for coaches given that 81.3% of the coaches surveyed reported an average annual income derived from their coach role of <10,000 euros.

Coaches have a key role in doping prevention (Kirby et al., 2011), but they can also represent a risk factor regarding doping use in their athletes as they may encourage them to dope or supply them with banned PESM (Allen et al., 2017; Vakhitova and Bell, 2018). In our study, the percentage of self-reported doping administration was 4.5% among coaches. Despite the lack of studies analyzing doping prevalence in coaches in the current literature, Morente-Sánchez and Zabala (2015) reported that 8.1% of the Spanish soccer coaches analyzed in their study used banned substances while a literature review (Backhouse and McKenna, 2012) indicated that 4–6% of the coaches and rest of ASP analyzed reported personal use of banned substances. Moreover, doping in sport is a prohibited and socially rejected practice, methods for assessing doping prevalence remain unclear and therefore data prevalence is potentially underestimated due to the sensitivity of the question. The prevalence of doping in elite sports is likely to be between 14 and 39% (De Hon et al., 2015). A recent meta-analysis study shows a disparate range between 0 and 73% (Gleaves et al., 2021) where authors suggest best practice recommendations and guidelines to improve the evidence quality in this field. The use of multiple measures to triangulate doping prevalence data, and indirect measures like randomized response technique (RRT) may provide more reliable measurements (Gleaves et al., 2021).

Overall, adaptation and application of the SDCM in ASP proves its reproducibility in other population. Our results show that moral disengagement, social norms, and motivational profiles are the strongest predictors of attitudes toward doping among coaches and 4.5% of them supply their athletes with prohibited substances or methods. Therefore, it is necessary to address these psychological-, attitude-, and behavior-related issues through educational programs targeting coaches. Furthermore, sanctions applied to coaches and the rest of professionals conforming the ASP seem scarce (World Anti-Doping Agency, 2020), probably due to the non-analytical nature of these ADRV, since it is difficult to prove their doping behaviors and therefore to sanction them. In addition, most of the social science research literature on doping in sport has focused on athletes, while coach-centered studies remain limited (Backhouse et al., 2015; García-Grimau et al., 2020). The results of the present study alongside those from others reveal that coaches tend to morally disengage through a lack of commitment and a diffusion of their responsibilities as educators in doping prevention (Barkoukis et al., 2019), and consider that they do not have adequate tools to prevent their athletes from doping use, while being aware of their role as antidoping educators though (Engelberg et al., 2019; Patterson et al., 2019). All this scientific evidence paints a worrying picture, as coaches could rather represent a doping risk. On the one hand, the threshold prevalence of doping among coaches is considerable (i.e., between 4.5 and 8.1%). On the second hand, they manifested an absence and lack of interest in doping prevention. In the complex context of elite sport in which the influences of sport environment and reference group on athletes are crucial, the absence of doping prevention may involve the presence of risk of its use. Perhaps it is time to focus more efforts on coaches, without putting aside the athletes, and therefore turn coaches into reliable doping prevention factors. To this end, it is necessary to enhance scientific research and then develop, implement, and promote more educational programs targeting coaches, on a mandatory basis while covering the specific needs of coaches so that they can perform their role as anti-doping educators in an effective, committed, and proactive manner.

### Limitations and Future Research

The participants in our study declared that they would not obtain any economic benefit if their athletes doped, and have low economic income derived from their activity as coaches. However, benefit appraisal did not influence their attitudes toward doping and it would not necessarily be the case in other cultural and social contexts. Reproducing this study in other countries would allow us to elucidate the specific impact of this variable and others influencing doping attitudes and behaviors in coaches. More specifically, further studies examining in depth the relationship among moral variables, descriptive norms, motivational and psychological profiles, and doping attitudes in coaches from other cultural contexts are encouraged. In addition, further studies analyzing the effectiveness of interventions aiming at avoiding moral disengagement in coaches are recommended, and it would be useful to analyze other sociodemographic variables in coaches, as for example, if they were competitive athletes. The model explaining attitudes toward doping in coaches seems to be less complex than that in athletes given that it displays fewer influencing factors. Doping prevalence among coaches was measured in terms of self-reported administration or attempted administration to athletes of a banned PESM, but the six remaining ADRVs included in the Code of the World Anti-Doping Agency (2021a) were not tested in the present study. In addition to the limitation of the bias response in self-reported doping, outcome of doping prevalence may be underestimated.

## Conclusion

Attitudes toward the use of doping in Spanish track and field national coaches were analyzed for the first time to the best of authors' knowledge through the SDCM adaptation for coaches, which displayed reproducibility. Moral disengagement, social norms, and motivational profiles were the strongest predictors of positive attitudes toward doping among these coaches. Accordingly, further design of anti-doping research and education should target on developing and improving these abilities in coaches. It is necessary to enhance coach-centered research, provide more assistance to sport coaches, and establish effective and mandatory anti-doping education in them.

## Data Availability Statement

The datasets presented in this article are not readily available for ethical reasons according to an explicit condition set by the Ethics Committee from the World Anti-Doping Agency (2019-A2). Requests to access the datasets should be directed to the corresponding author.

## Ethics Statement

The studies involving human participants were reviewed and approved by Isabel I de Castilla International University (UI1-PI016) World Anti-Doping Agency (2019-A2). The patients/participants provided their written informed consent to participate in this study.

## Author Contributions

EG-G: designed, conceptualized, performed analysis, and wrote the paper. EG-G and AC: collected the data. AC and RD: contributed to the writing. All authors contributed to the article and approved the submitted version.

## Funding

This work was funded by the World Anti-Doping Agency, within their Social Science Research Grants program and Isabel I de Castilla International University.

## Conflict of Interest

The authors declare that the research was conducted in the absence of any commercial or financial relationships that could be construed as a potential conflict of interest.

## Publisher's Note

All claims expressed in this article are solely those of the authors and do not necessarily represent those of their affiliated organizations, or those of the publisher, the editors and the reviewers. Any product that may be evaluated in this article, or claim that may be made by its manufacturer, is not guaranteed or endorsed by the publisher.
